# Intraepithelial serous carcinoma of the endometrium: protocol for a prospective multicentre registry in the Netherlands

**DOI:** 10.1136/bmjopen-2026-120737

**Published:** 2026-08-07

**Authors:** F Ciska Slaager, Klaas J Hoogduin, Ward Hofhuis, Floris H Groenendijk, Heleen J van Beekhuizen

**Affiliations:** 1 Gynaecological Oncology, Erasmus MC University Medical Center Rotterdam, Rotterdam, The Netherlands; 2 Department of Pathology, Pathan BV, Rotterdam, The Netherlands; 3 Department of Gynaecology, Franciscus Gasthuis en Vlietland, Rotterdam, The Netherlands; 4 Pathology, Erasmus MC Cancer Center, Rotterdam, The Netherlands; 5 Department of Obstetrics and Gynaecology, Erasmus MC Cancer Center, Rotterdam, The Netherlands

**Keywords:** Cancer, Gynaecological oncology, Histopathology

## Abstract

**Abstract:**

**Introduction:**

Serous endometrial intraepithelial carcinoma (SEIC) is a rare, non-invasive lesion of the endometrial epithelium, typically characterised by p53 abnormalities. Although regarded as a precursor lesion, recent classification systems increasingly position SEIC within the broader spectrum of serous carcinomas and current guidelines place non-invasive p53-abnormal serous lesions in an uncertain risk category due to limited outcome data. As a result, SEIC is not consistently addressed as a distinct diagnosis, contributing to variation in clinical management. Complete surgical staging, including pelvic and para-aortic lymph node assessment, is frequently performed, yet supporting evidence remains scarce. This study aims to collect longitudinal data to evaluate surgical management strategies in relation to clinical outcomes, survival and quality of life.

**Methods and analysis:**

This multicentre prospective observational cohort will enrol patients diagnosed with SEIC or equivalent non-invasive serous lesions who provide informed consent. Data will be collected using electronic case report forms (Castor Electronic Data Capture). Baseline clinical, pathological, molecular and treatment data will be collected, including imaging, histopathology, immunohistochemistry and molecular classification when available. Patient-reported outcomes will be assessed using the European Organisation for Research and Treatment of Cancer Quality of Life Questionnaire Core 30 (EORTC QLQ-C30), European Organisation for Research and Treatment of Cancer Quality of Life Questionnaire Endometrial Cancer Module (EORTC QLQ-EN24) and EuroQol 5-Dimension 5-Level questionnaire (EQ-5D-5L) questionnaires at baseline, 6 months, 2 years and 5 years after diagnosis. The primary outcome is progression-free survival, secondary outcomes include overall survival, health-related quality of life and adverse events. Analyses will be primarily descriptive. Survival outcomes will be estimated using Kaplan-Meier methods, with Cox proportional hazards modelling used to explore predictors of outcome where feasible.

**Ethics and dissemination:**

The study will be conducted in accordance with the Declaration of Helsinki and applicable regulations. Ethical approval was obtained at the Erasmus MC (MEC-2025-0368). Results will be disseminated through peer-reviewed publications and scientific meetings.

**Trial registration number:**

NCT06677242.

**STRENGTHS AND LIMITATIONS OF THIS STUDY**

- This prospective multicentre registry is specifically designed for patients with serous endometrial intraepithelial carcinoma (SEIC) and equivalent non-invasive serous endometrial lesions.
- Standardised collection of clinical, pathological, molecular and patient-reported outcome data enables comprehensive long-term follow-up.
- The observational design reflects real-world clinical practice across academic and non-academic centres.
- Owing to the rarity of SEIC, the anticipated sample size is limited, which may restrict statistical power for subgroup and multivariable analyses.
- Central pathology review is strongly recommended but not mandatory, and treatment allocation is not protocol-mandated, introducing potential diagnostic heterogeneity and confounding by indication.

## Introduction

 Serous endometrial intraepithelial carcinoma (SEIC) (Tumor characteristics were coded according to the International Classification of Diseases for Oncology (ICD-O) code 8441/2) is defined as a non-invasive lesion of the endometrial epithelium, replacing the native surface of the endometrium.^1 2^ A p53 mutation is often present, diagnosed through immunohistochemistry, which classifies SEIC in the p53 mutant endometrial tumours.^3 4^ Despite its non-invasive nature, SEIC exhibits malignant potential and poor prognosis, with documented cases of extrauterine dissemination even at presentation.^5–8^ Published case series and a recently published review have recommended performing surgical staging as primary treatment.^1 7 9–12^ In the Netherlands, clinical management varies from therapeutic hysteroscopy to surgical staging procedures.^13^ International guidelines lack specific recommendations for managing SEIC as a distinct entity. Although SEIC is currently defined as a non-invasive serous lesion, it is increasingly considered part of the serous, p53-mutated endometrial tumours rather than as a distinct entity.^12 14 15^ The collaborative guideline for managing patients with endometrial carcinoma by the European Society of Gynaecological Oncology, European Society for Radiotherapy and Oncology, and European Society of Pathology positions SEIC-equivalent FIGO IC (stage IC according to the International Federation of Gynecology and Obstetrics staging system) serous lesions in a grey zone of ‘uncertain risk’ due to insufficient outcome data, underscoring the lack of consensus and the evolving nature of its classification. This guideline has no explicit management recommendations for the uncertain risk category.^15^ The National Comprehensive Cancer Network guideline in the USA advises surgical staging and adjuvant therapy for all serous carcinoma FIGO 1A or higher, without a distinct section dedicated to SEIC management.^16 17^ Surgical staging, whether performed via laparotomy or minimally invasive techniques, entails a comprehensive procedure associated with recognised risks of complications.^18 19^ The impact of surgical staging on SEIC survival outcomes remains uncertain due to limited data. Lymph node sampling, especially lymph node dissection, lacks clear evidence, leaving ambiguity about SEIC metastasis to pelvic or para-aortic/paracaval nodes. Bilateral sentinel lymph node sampling has also been recommended, as sentinel lymph node procedures are increasingly performed for endometrial carcinomas.^12^ We propose that superficial intraepithelial carcinoma may disseminate via the fallopian tubes into the peritoneal cavity. Consequently, the necessity of lymph node sampling or dissection in SEIC treatment remains uncertain. In our recently published retrospective cohort study, 23 patients diagnosed with SEIC were evaluated, revealing no evidence of disease dissemination at the time of surgery. Over a follow-up period of nearly 3 years, no disease recurrences have been documented.^20^ Since complete staging did not detect any metastasis in this study it might be safe to omit standard staging procedure and to perform a simple hysterectomy with peritoneal washings and peritoneal biopsies (including biopsy of the infracolic omentum). Therefore, the primary objective of this study is to establish an international registry and longitudinally monitor patients diagnosed with SEIC, thereby contributing to the augmentation of knowledge and improvement of treatment strategies. This study establishes a registry for longitudinally monitoring of patients with non-invasive serous carcinomas/SEIC. By evaluating surgical and systemic treatments, we aim to enhance knowledge and refine treatment strategies, ultimately impacting patient survival outcomes and quality of life (QoL). Because the terminology may be further consolidated in the coming years, this study uses the combined term ‘non-invasive serous carcinoma’ including SEIC and FIGO IC serous carcinomas, and includes all lesions meeting the pathological criteria for SEIC, regardless of diagnostic nomenclature. This ensures compatibility with current and future classification systems. We hypothesise that patients with non-invasive serous carcinoma of the endometrium have a low risk of extrauterine disease and recurrence, and that less extensive surgical management may be associated with similar oncological outcomes while potentially reducing treatment-related morbidity and preserving QoL. This registry is designed to explore this hypothesis and generate evidence for future studies.

## Methods and analysis

### Study objectives

The aims of this registration study include: (1) To establish and maintain a prospective observational registry of patients diagnosed with SEIC and equivalent non-invasive serous tumours. (2) To systematically collect clinical, diagnostic, tumour, treatment and outcome data in patients with SEIC. (3) To evaluate the association between the extent of surgical treatment, the use of adjuvant therapy and progression-free survival (PFS).

### Study design

This is an observational cohort study conducted in the Netherlands, with the intention to expand into an international, multicentre registry. International centres with expertise in gynaecological oncology will be invited to participate using the same data collection framework. The estimated duration of the registration is 10 years: inclusion of participants during 5 years with a target follow-up duration of 5 years per participant. Regarding therapy of SEIC, all options are allowed (surgery, systemic therapy, radiotherapy). All interventions are part of regular medical treatment and are registered. This is an observational study; no interventions are performed.

### Study population

#### Inclusion criteria

Participants are eligible for inclusion if they meet all of the following criteria:

1. Histopathologically confirmed serous intraepithelial endometrial lesion, defined as:
  - Serous morphology consistent with SEIC, including p53-abnormal immunophenotype and high-grade nuclear atypia.
  - No evidence of stromal or myometrial invasion on histopathologic evaluation.
  - Diagnosed using any of the following accepted diagnostic labels:
    - SEIC.
    - FIGO IC serous carcinoma.
    - Serous carcinoma in situ.
    - Minimal uterine serous carcinoma.
2. ≥18 years of age.
3. Signed informed consent.

#### Exclusion criteria

Participants are not eligible for inclusion if they meet one of the following criteria:

1. Any diagnosis other than a non-invasive serous intraepithelial endometrial lesion, including:
  - Endometrioid carcinoma.
  - Clear-cell carcinoma.
  - Serous carcinoma with invasion.
  - Carcinosarcoma.
  - Endometrial hyperplasia or endometrial intraepithelial neoplasia.
2. Cases in which histopathology shows (also) myometrial or stromal invasion, even if previously labelled SEIC.

#### Case identification and recruitment

Patients will be enrolled by the principal investigator, the coordinating investigator or the research nurse of the Erasmus Medical Center, after signed informed consent was obtained at one of the participating centres. Patients will be informed by their primary clinician (gynaecologist) and provided with a patient information brochure. If the patient is interested in participating, a hardcopy informed consent form is provided or a link to ValidSign^21^ via email will be sent, where a special PDF version of the informed consent form is available. The participant can sign this with an ‘advanced electronic signature’ with SMS verification. The digital consent form will then be downloaded to a secure drive on the Erasmus MC network for archiving. From all participants who provided written informed consent in a participating centre, the signed form will be scanned and stored digitally in the participating centre. All clinical data are prospectively collected through electronic case report forms (e-CRF) by the treating gynaecologist of the participating centre. The coordinating investigator will re-evaluate the provided data and ensure that collecting data is complete. All participants will receive an online questionnaire using a survey package in Castor Electronic Data Capture (EDC) at the time of diagnosis and during follow-up after 6 months, 2 years and 5 years of diagnosis, respectively. The survey package includes three online questionnaires: EQ-5D-5L QOL, QLQ-EN24 and QOL-C30. The estimated time needed to complete the three questionnaires is 15 min. Regarding therapy of SEIC, all treatment options are allowed (surgery, systemic therapy, radiotherapy). There will be no specific, patient-tailored recommendations regarding the given therapy. All interventions are part of regular medical treatment and are registered. The methods are all part of standard clinical care, apart from the collection of data through the questionnaires. Since this is an observational study, no interventions are performed.

#### Sample size calculation

A formal sample size or power calculation was not performed because this study is designed as a prospective registry of a rare disease rather than a hypothesis-testing clinical trial. The primary objective is to establish a comprehensive longitudinal registry and include as many eligible patients as possible to improve understanding of this rare disease entity. In a previously cited retrospective study conducted in the Netherlands, 23 patients were diagnosed with SEIC over an 8-year period (2012–2020).^20^ The Netherlands Comprehensive Cancer Organisation (IKNL) registered 77 patients with SEIC between 2015 and 2025. We estimate that more patients may be diagnosed in the coming years due to increased awareness among gynaecologists and pathologists and improved recognition of the disease entity. Additionally, increased awareness may lead to more frequent resection of endometrial polyps and subsequent diagnosis of SEIC. Therefore, we anticipate approximately 100 eligible patients in the Netherlands, of whom approximately 75% may consent to participate in the registry. Participation of centres outside the Netherlands is expected to further increase the number of eligible patients. The aim is to include all eligible patients diagnosed during the study period to maximise the completeness of data collection and improve the precision of descriptive estimates. Data generated through this registry are expected to support future hypothesis-driven studies and inform clinical management of this rare disease entity.

### Outcomes

#### Primary and secondary outcome

The primary outcome is PFS, defined as the time from histopathological diagnosis of the lesion to the first occurrence of disease progression, recurrence or death from any cause, whichever occurs first. Disease progression or recurrence is defined as radiologically, histologically, or clinically documented local, regional or distant disease. Patients without an event will be censored at the date of last follow-up. Secondary outcomes include overall survival (OS), disease-related symptoms, health-related QoL, type and extent of surgical and adjuvant treatment, severe postoperative complications (Clavien-Dindo grade III–IV), and treatment-related adverse events associated with systemic therapy or radiotherapy classified according to the Common Terminology Criteria for Adverse Events (CTCAE) version 5.0.^22 23^ OS will be defined as the time from histopathological diagnosis to death from any cause. Study outcomes/endpoints include the baseline characteristics of patients, diagnostic measurements, treatment details and follow-up data.

### Data collection and data management

#### Data collection

All eligible patients who provide informed consent will be included in a secure online database using Castor EDC. Clinical data will be collected through e-CRFs completed by the treating gynaecologist at the participating centre. The coordinating investigator will review all entered data to ensure completeness and consistency. Participants will complete validated online questionnaires via Castor EDC at baseline (prior to treatment, if applicable), and at 6 months, 2 years and 5 years after diagnosis.

#### Collected variables

Baseline characteristics include age, body mass index, type of physical complaints, medical history, use of medication and Eastern Cooperative Oncology Group (ECOG) performance status (known as WHO performance score).^24^ Diagnostic measurements include imaging details (date of imaging, results of gynaecological examination and advanced imaging), biochemical laboratory results and histopathology results of endometrial biopsies and surgical specimens. Regarding the histopathological examination, the following data will be obtained: the histomorphological characteristics, immunohistochemistry staining on mismatch repair proteins (MMR) and p53 mutation and results of molecular diagnostics (MSI test, p53 mutation analysis, POLE mutation analysis). Treatment details include details on the surgical treatment (date, type of surgical procedure (hysteroscopy, hysterectomy with/without salpingo-oophorectomy, staging procedure, cytoreductive surgery), perioperative findings (dissemination of disease and FIGO stage of disease), and perioperative complications) and details on administered adjuvant therapy (date, duration and type/regimen of adjuvant therapy). Given anticipated changes in FIGO and WHO terminology, all cases will be stored with raw histopathology and molecular data, allowing for retrospective reclassification according to future systems. Follow-up data will be collected at 6 months, 2 years and 5 years after diagnosis and include clinical status, symptoms, QoL, evidence of disease recurrence and disease-related or unrelated death.

#### Data management

This research protocol adheres to the principles and requirements of the Dutch implementation of the EU General Data Protection Regulation (GDPR). All data collected during the study will be processed lawfully, fairly and transparently and limited to what is necessary to achieve the study objectives.

#### Data storage and access control

All study data will be stored in Castor EDC, a GDPR-compliant EDC system. Each participant will be assigned a unique study identification code that is not derived from personal identifiers. The key linking study IDs to patient identities will be stored securely at the participating centre and safeguarded by the local investigator. The coordinating investigator and principal investigator are authorised to enrol participants, manage the e-CRFs and export pseudonymised data for analysis. Referring gynaecologists are authorised only to complete patient-specific e-CRFs for participants from their centre. In their absence, a delegated physician from the same medical centre may complete the e-CRF. Pseudonymised datasets will be exported to Excel and SPSS for statistical analysis. Pseudonymised research data from e-CRFs and questionnaires will be stored on a secure, institutionally managed online drive with restricted access for members of the SEIC Study Group.

#### Data sharing

After completion of the primary analyses, anonymised datasets may be shared with collaborating research centres on reasonable request and approval by the study investigators, in accordance with applicable data-protection regulations.

#### Pathology data and biological material

In the Netherlands, pathology data are registered in the Dutch Nationwide Pathology Databank (PALGA). Pathology results from referring centres will be entered into the e-CRF. Access to PALGA’s internal database by members of the research team will only occur if participants have provided explicit consent and if necessary for completeness of data. For international participating centres, e-CRFs will be completed by the treating clinician or pseudonymised pathology reports will be securely transmitted to the coordinating investigator. Additional diagnostic analyses (eg, immunohistochemistry, mutation analysis, MSI testing) may be performed if not previously available. Results and images will then be stored in the secure internal pathology database of Erasmus MC. Pathology specimens are coded using original pathology (T-)codes and stored in accordance with routine clinical practice for a minimum of 10 years. Use of residual material for additional analysis will only occur if participants provide consent. Participating centres are strongly encouraged to involve pathologists with expertise in gynaecological pathology. Whenever feasible, cases will undergo review by a gynaecological pathologist with expertise in serous endometrial neoplasia. If this expertise is not available in the participating centre or within the regional gynaecological oncology network, central pathology review will be performed by the coordinating research team of the Erasmus Medical Center. Any diagnostic revisions following expert review will be documented in the registry. Furthermore, raw histopathological and molecular data will be collected to facilitate reassessment and future reclassification where necessary. The T-code is not directly linked to hospital patient identifiers and can only be linked to study IDs by authorised pathologists.

#### Withdrawal and data retention

If a participant withdraws from the study or is lost to follow-up, the reason will be recorded in the e-CRF. Data collected up to the point of withdrawal may be retained and analysed unless the participant explicitly requests deletion, in accordance with GDPR. Study data will be stored for a minimum of 15 years after study completion.

### Statistical analysis

Baseline characteristics, treatment details and outcome measures will be analysed using descriptive statistics. Continuous variables will be summarised using means and SD or medians and IQRs, as appropriate. Categorical variables will be presented as frequencies and percentages. Longitudinal QoL scores will be summarised descriptively over time. Comparisons between groups, if applicable, will be performed using the χ² test or Fisher’s exact test for categorical variables and the unpaired T-test or Mann-Whitney U test for continuous variables, depending on data distribution. If subgroup analyses are feasible, patients will be stratified according to the extent of surgical treatment (eg, complete surgical staging vs hysterectomy with bilateral salpingo-oophorectomy only). PFS and OS will be estimated using the Kaplan-Meier method, and differences between groups will be assessed using the log-rank test. Survival analyses will use the date of histopathological diagnosis as the index date. Univariate Cox proportional hazard regression analyses will be performed to explore potential predictors of survival outcomes. Multivariable Cox regression analysis will be conducted only if the number of events allows reliable modelling. Results will be reported as HRs with 95% CIs. All statistical analyses will be performed using the SPSS, V.25.0. A two-sided p<0.05 will be considered statistically significant.

### Missing data

The extent and patterns of missing data will be assessed for all key variables. Variables with more than 5% missing observations will be reported explicitly. For EORTC questionnaires (QLQ-C30 and QLQ-EN24), missing items will be handled according to the EORTC scoring manuals. Scale scores will be calculated when at least half of the items contributing to a scale have been completed; missing items within eligible scales will be replaced by the mean of the completed items from that scale. If fewer than half of the items are completed, the scale score will be considered missing. Complete-case analyses will be used for primary analyses. Additional sensitivity analyses may be considered if the extent of missing data is substantial and the number of observations permits.

### Study status

Participant recruitment started in May 2026 and is expected to be completed in April 2031. On 1 February 2026, 10 participating centres are involved. We are open to more collaborations, nationally and internationally.

## Ethics and dissemination

Central study approval was obtained at the Erasmus MC (MEC-2025-0368). Results of this study will be published in peer-reviewed journals and will be shared in reports of relevant conferences and congresses. All principal investigators of participating centres are joined in the SEIC Study Group, to share expertise on this topic. Researchers contributing to study conceptualisation, design, data collection, analysis and writing or reviewing of the manuscript are listed as authors.

### Patient and public involvement

Patients or the public are not directly involved in the design of the study or its implementation.

## Supplementary material

10.1136/bmjopen-2026-120737 online supplemental file 1
