# Supplementary material for: Intraepithelial serous carcinoma of the endometrium: protocol for a prospective multicentre registry in the Netherlands
Source: BMJ Open. 2026 Aug 7;16(8):e120737. doi: 10.1136/bmjopen-2026-120737 (PMC13475400; doi:10.1136/bmjopen-2026-120737)
Supplement: Supplementary file 1 [file bmjopen-2026-120737.reviewer_comments.pdf]

PEER REVIEW HISTORY

BMJ Open publishes all reviews undertaken for accepted manuscripts. Reviewers are asked to complete a checklist review form and are provided with free text boxes to elaborate on their assessment. These free text comments are reproduced below.

ARTICLE DETAILS

Title (Provisional)

Intraepithelial Serous Carcinoma of the Endometrium: Protocol for a Prospective Multicentre Registry in The Netherlands.

Authors

Slaager, F. Ciska; Hoogduin, Klaas J; Hofhuis, Ward; Groenendijk, Floris H; van Beekhuizen , H J

VERSION 1 - REVIEW

**Reviewer**	**1**
**Name**	**Hoegl, Jorge**
**Affiliation**	**Centro Clinico Profesional, Gyne Onc**
**Date**	**24-Jun-2026**
**COI**	**None**

The protocol addresses a relevant clinical gap in an uncommon condition. Its overall structure is appropriate for an observational registry. The following suggestions and improvements are recommended by the section.

Title and abstract

In the “Strengths and limitations” box, only a single descriptive statement is provided. This section should be expanded to include at least three genuine limitations, such as the absence of mandatory central pathology review, the lack of a formal sample size calculation, and the observational nature of the study without a control group.

Introduction

The working hypothesis is not stated. The protocol should formulate a clear and explicit hypothesis at the end of the introduction. Without this, the design lacks analytical direction.

Methods

The protocol states that review by a pathologist with gynecologic expertise is “highly recommended” and “performed when feasible.” Since SEIC is a diagnosis defined exclusively by histopathologic and immunohistochemical criteria, including p53 pattern, nuclear atypia,

and absence of invasion, the lack of mandatory central review may introduce significant bias. It should also be clarified whether the participating pathologists are specifically familiar with this disease entity.

The index date is defined either as the date of surgery or, if surgery is not performed, the date of histopathologic diagnosis. Prior diagnostic hysteroscopy before definitive hysterectomy introduces ambiguity in this definition.

I suggest establishing a single, reproducible index date: either the date of definitive surgery or, alternatively, the date of the biopsy that led to the diagnosis, provided that this choice is defined and applied uniformly throughout the protocol.

Only the Clavien-Dindo classification is mentioned for surgical complications. Patients receiving adjuvant treatment, such as chemotherapy or radiotherapy, also require systematic recording of systemic toxicity. I recommend adding the CTCAE classification (version 5.0) for non-surgical adverse events.

The handling of missing data is limited to "complete-case analysis." This approach is insufficient when missingness is expected in quality-of-life questionnaires and clinical variables. I recommend specifying a threshold for reporting missing data and detailing how missing items in the EORTC questionnaires will be managed, in accordance with the scoring manual.

VERSION 1 - AUTHOR RESPONSE

Reviewer's comments

1. *In the "Strengths and limitations" box, only a single descriptive statement is provided. This section should be expanded to include at least three genuine limitations, such as the absence of mandatory central pathology review, the lack of a formal sample size calculation, and the observational nature of the study without a control group.*
   Response: We thank the reviewer for this suggestion and agree that the Strengths and Limitations section should provide a more balanced overview of the study design. We have revised this section to include additional limitations related to the observational design, anticipated sample size, and absence of mandatory central pathology review. In addition, we have further clarified in the Sample Size section why a formal sample size calculation was not performed.
   Revision:
   a. The Strengths and Limitations box has been expanded to include:
      - This prospective multicentre registry is specifically designed for patients with serous endometrial intraepithelial carcinoma (SEIC) and equivalent non-invasive serous endometrial lesions.
      - Standardised collection of clinical, pathological, molecular and patient-reported outcome data enables comprehensive long-term follow-up.
      - The observational design reflects real-world clinical practice across academic and non-academic centres.

    - Owing to the rarity of SEIC, the anticipated sample size is limited, which may restrict statistical power for subgroup and multivariable analyses.
    - Central pathology review is strongly recommended but not mandatory, and treatment allocation is not protocol-mandated, introducing potential diagnostic heterogeneity and confounding by indication.
  b. The Sample Size section has been revised to clarify why a formal sample size calculation was not performed: "A formal sample size or power calculation was not performed because this study is designed as a prospective registry of a rare disease rather than a hypothesis-testing clinical trial. The primary objective is to establish a comprehensive longitudinal registry and include as many eligible patients as possible to improve understanding of this rare disease entity. In a previously cited retrospective study conducted in the Netherlands, 23 patients were diagnosed with SEIC over an eight-year period (2012–2020). The Netherlands Comprehensive Cancer Organisation (IKNL) registered 77 patients with SEIC between 2015 and 2025. We estimate that more patients may be diagnosed in the coming years due to increased awareness among gynaecologists and pathologists and improved recognition of the disease entity. Additionally, increased awareness may lead to more frequent resection of endometrial polyps and subsequent diagnosis of SEIC. Therefore, we anticipate approximately 100 eligible patients in the Netherlands, of whom approximately 75% may consent to participate in the registry. Participation of centres outside the Netherlands is expected to further increase the number of eligible patients. The aim is to include all eligible patients diagnosed during the study period to maximise the completeness of data collection and improve the precision of descriptive estimates. Data generated through this registry are expected to support future hypothesis-driven studies and inform clinical management of this rare disease entity.''

2. *The working hypothesis is not stated. The protocol should formulate a clear and explicit hypothesis at the end of the introduction. Without this, the design lacks analytical direction.*
   Response: We appreciate this comment and have added a study hypothesis at the end of the Introduction. As this is a prospective registry designed primarily to generate evidence in a rare disease, we formulated the hypothesis cautiously and consistently with the exploratory nature of the study.
   Revision: The following text has been added at the end of the Introduction: "We hypothesise that patients with non-invasive serous carcinoma of the endometrium have a low risk of extrauterine disease and recurrence, and that less extensive surgical management may be associated with similar oncological outcomes while potentially reducing treatment-related morbidity and preserving quality of life. This registry is designed to explore this hypothesis and generate evidence for future studies."

3. *The protocol states that review by a pathologist with gynaecologic expertise is "highly recommended" and "performed when feasible." Since SEIC is a diagnosis defined exclusively by histopathologic and immunohistochemical criteria, including p53 patterns, nuclear atypia, and absence of invasion, the lack of mandatory central review may introduce significant bias. It should also be clarified whether the participating pathologists are specifically familiar with this disease entity.*

Response: We thank the reviewer for this important comment and agree that diagnostic consistency is crucial for this rare and evolving disease entity. In participating centres within the Netherlands, review by a pathologist with expertise in gynaecological pathology is strongly recommended and is generally feasible through local expertise or consultation within regional gynaecological oncology networks. However, because the registry is designed to expand internationally, mandatory central pathology review for all cases may not always be feasible. To maximise diagnostic accuracy and consistency, we have clarified the expected level of pathology expertise and expanded the description of pathology review procedures. In addition, detailed histopathological and molecular data will be collected to facilitate diagnostic verification, future reassessment, and reclassification where necessary.
Revision: The manuscript now states: "Participating centres are strongly encouraged to involve pathologists with expertise in gynaecological pathology. Whenever feasible, cases will undergo review by a gynaecological pathologist with expertise in serous endometrial neoplasia. If this expertise is not available in the participating center, central pathology review will be performed by the coordinating research team of the Erasmus Medical Center. Any diagnostic revisions following expert review will be documented in the registry. Furthermore, raw histopathological and molecular data will be collected to facilitate reassessment and future reclassification where necessary."

4. *The index date is defined either as the date of surgery or, if surgery is not performed, the date of histopathologic diagnosis. Prior diagnostic hysteroscopy before definitive hysterectomy introduces ambiguity in this definition. I suggest establishing a single, reproducible index date.*
   Response: We thank the reviewer for identifying this ambiguity. To ensure a single, reproducible index date applicable to all participants and to avoid ambiguity arising from diagnostic procedures preceding definitive treatment, the date of histopathological diagnosis will be used as the index date for all time-to-event analyses.
   Revision: The definition of progression-free survival has been revised as follows: "Progression-free survival (PFS) is defined as the time from histopathological diagnosis of the lesion to the first occurrence of disease progression, recurrence, or death from any cause, whichever occurs first." A similar revision has been applied throughout the manuscript wherever the index date was referenced.

5. *Only the Clavien-Dindo classification is mentioned for surgical complications. Patients receiving adjuvant treatment, such as chemotherapy or radiotherapy, also require systematic recording of systemic toxicity. I recommend adding the CTCAE classification (version 5.0) for non-surgical adverse events.*
   Response: We fully agree and have incorporated systematic registration of treatment-related toxicity using CTCAE version 5.0.
   Revision: The Outcomes section has been revised as follows: ''Secondary outcomes include overall survival, disease-related symptoms, health-related quality of life, type and extent of surgical and adjuvant treatment, severe postoperative complications (Clavien-Dindo grade III–IV), and treatment-related adverse events associated with systemic therapy or radiotherapy classified according to the Common Terminology Criteria for Adverse Events (CTCAE) version 5.0.''

6. *The handling of missing data is limited to "complete-case analysis." This approach is insufficient when missingness is expected in quality-of-life questionnaires and clinical variables. I recommend specifying a threshold for reporting missing data and detailing how missing items in the EORTC questionnaires will be managed.*
   Response: We thank the reviewer for this helpful suggestion and agree that the handling of missing data should be described in greater detail, particularly for patient-reported outcome measures. We have expanded the statistical analysis section to specify how missing questionnaire items will be handled according to the EORTC scoring manuals and how missing data in other study variables will be reported and evaluated.
   Revision: We added a separate missing data section below the section on statistical analysis: ''The extent and patterns of missing data will be assessed for all key variables. Variables with more than 5% missing observations will be reported explicitly. For EORTC questionnaires (QLQ-C30 and QLQ-EN24), missing items will be handled according to the EORTC scoring manuals. Scale scores will be calculated when at least half of the items contributing to a scale have been completed; missing items within eligible scales will be replaced by the mean of the completed items from that scale. If fewer than half of the items are completed, the scale score will be considered missing. Complete-case analyses will be used for primary analyses. Additional sensitivity analyses may be considered if the extent of missing data is substantial and the number of observations permits.''
