# Supplementary material for: Intraepithelial serous carcinoma of the endometrium: protocol for a prospective multicentre registry in the Netherlands
Source: BMJ Open. 2026 Aug 7;16(8):e120737. doi: 10.1136/bmjopen-2026-120737 (PMC13475400; doi:10.1136/bmjopen-2026-120737)
Supplement: Supplementary file 2 [file bmjopen-16-8-s001.docx]

**Appendix I	List of collaborators and study sites**

| J.M.A. Pijnenborg | Department of Gynaecological Oncology, Radboud University Medical Center, Nijmegen, The Netherlands. |
| --- | --- |
| M. Rijken, J.W. Trum | Department of Gynaecological Oncology, Antoni van Leeuwenhoek Hospital, Netherlands Cancer Institute, Amsterdam, The Netherlands. |
| A. Baalbergen | Department of Gynaecology, Reinier de Graaf Gasthuis, Delft, The Netherlands. |
| J.M.J. Piek | Department of Gynaecological Oncology, Catharina Ziekenhuis, Eindhoven, The Netherlands. |
| C.G. Gerestein | Department of Gynaecological Oncology, University Medical Center Utrecht, The Netherlands. |
| H.P.M. Smedts | Department of Gynaecology, Amphia ziekenhuis, Breda, The Netherlands. |
| P.M.L.H. Vencken, M.Z. Dorman | Department of Gynaecology, Bravis ziekenhuis, Bergen op Zoom, The Netherlands. |
| P.J. de Vos van Steenwijk | Department of Gynaecological Oncology, Maastricht University Medical Center, Maastricht, The Netherlands |
| W. Hofhuis | Department of Gynaecology, Franciscus Gasthuis & Vlietland, Rotterdam, The Netherlands |
| G.M. Nieuwenhuyzen-de Boer, A.S. Tjalsma | Department of Gynaecology, Albert Schweitzer ziekenhuis, Dordrecht, The Netherlands |
